# Supplementary material for: Assessing the response of micro-eukaryotic diversity to the Great Acceleration using lake sedimentary DNA
Source: Nat Commun. 2020 Jul 31;11:3831. doi: 10.1038/s41467-020-17682-8 (PMC7395174; doi:10.1038/s41467-020-17682-8)
Supplement: Supplementary file 1 — Supplementary Information [file 41467_2020_17682_MOESM1_ESM.pdf]

Supplementary Information for:

Assessing the response of micro-eukaryotic diversity to the Great Acceleration using lake sedimentary DNA

Keck et al.

Supplementary Information

Supplementary Methods . . . 3
Supplementary Figures . . . 9
Supplementary Figure 1. . . . 9
Supplementary Figure 2. . . . 10
Supplementary Figure 3. . . . 11
Supplementary Figure 4. . . . 12
Supplementary Figure 5. . . . 13
Supplementary Figure 6. . . . 14
Supplementary Figure 7. . . . 15
Supplementary Figure 8. . . . 16
Supplementary Figure 9. . . . 17
Supplementary Tables . . . 18
Supplementary Table 1. . . . 18
Supplementary Table 2. . . . 19
Supplementary Table 3. . . . 20
Supplementary Table 4. . . . 21
Supplementary Table 5. . . . 23
Supplementary References . . . 24

Supplementary Methods

General precautions taken for sedimentary DNA analysis

Various methodological precautions are required to achieve robust results for the analysis of paleo-environmental DNA.[1–3] We applied strict laboratory protocols to ensure the validity of our molecular data.

- Sterile disposable materials (labware, gloves, etc.) were used for all lab procedures; during sediment core subsampling, sediment slices were taken using sterilised metal plates.
- Separate stations were organized for subsampling, DNA extractions and PCR amplifications. To prevent contamination with modern DNA, the extraction of DNA from sediment is carried out in specific rooms dedicated to rare DNA, and PCR are prepared under designated working stations. These laboratories are physically separated from other molecular biology laboratories.
- Negative controls are included at 3 steps of the procedure: during subsampling (open tubes containing pure water), extraction (traitement of pure water) and PCR preparation (blank PCR tube). All blanks were found negative for the amplification of 18S rRNA markers; consequently the blanks were not included in the sequencing library.
- We selected short barcodes adapted to the work on sedimentary DNA. The choice of the barcode region was previously explained in Capo et al;[4] based on verifications of the coverage of primers for micro-eukaryotic diversity, the ability to amplify the DNA with these primers, and the quality of taxonomic assignment obtained, the probes 960F and NSR1438 were selected.
- To evaluate the potential influence of co-extracted inhibitors present in sedimentary DNA extracts (that may reduce the efficiency of downstream PCR), we assessed the inhibition level using quantitative PCR assays. The approach applied is based on the assumption that inhibitors are diluted out when a log-linear relationship is achieved between Cq and the dilution factor.[5] No inhibition effect was found.
- We performed a duplicate extraction for each strata and verified the similarity of DNA results obtained for the replicates of a given strata (Supplementary Figure 9).
- For one taxonomic group (Chlorophyceae) which was found to increase markedly in recent deposits, we verified the consistency between the percentage of DNA reads found by metabarcoding and the number of gene copies found by qPCR (qPCR assays using specific primers for chlorophyceae 18S rDNA according to Turon et al[6]). The correlation was found to be significant (correlation between sequenced reads of chlorophyceae and qPCR copies of chlorophyceae per gram of wet sediment: r=0.78, n=90).
- Data available from long term monitoring programs (survey of water quality), were used to explore

the consistency between data obtained here from sedimentary DNA and known trends reported by traditional monitoring (phytoplanktonic counts in the epilimnion). This was unfortunatly possible only for two lakes (Léman and Aiguebelette, and to a lesser extent Bourget) that have been monitored for ~40 years. The trends we report in particular for mixotrophs match with data from monitoring programs. Our observations were indeed consistent with the increase of Chrysophytes (Synura or Dinobryon) observed since the 1980s in lake Léman and lake Aiguebelette (results extracted from French technical reports produced by the Observatory on LAkes OLA; Rimet et al[7]). This type of comparison is however limited by the time period covered by traditionnal monitoring programs.

In compacted lake sediments, vertical advection of pore water is minimal, and multivalent metals and organic compounds (pigments, organic molecules with more than 15 carbon atoms) are immobilized in the sediment matrix.[8] Large organic molecules such as DNA are likely to adhere to solid-phase sediments (particles, particulate organic matter) or are locked in dead cells or ancient dormant resting cells. Therefore, leaching of DNA is unlikely to occur in lake sediment and lake sedimentary DNA is assumed to give an accurate temporal reconstruction of the biological community succession.[8–10]

The level of DNA preservation in the sediment (from one lake to another or when aging in sediment) is a sensitive point to be taken into account for paleo-reconstruction studies. Given the mechanisms of DNA protection by binding to mineral and organic particles and due to the absence of oxygen and UV radiation, aquatic sediments are, *a priori*, suitable environments for DNA preservation.[11,12] However, several processes can alter DNA sequences in marine[13,14] and freshwater sediments.[15,16] It was thus important to consider whether the differences observed between top and bottom strata could be induced by diagenetic processes responsible for the modification of DNA signal over time. Though shotgun sequencing allows to differentiate ancient DNA that has been damaged (typical damage patterns of ancient DNA marked by increase in T and A at the ends of DNA fragments), the limited number of samples that can be treated in parallel and the associated cost per sample still limit routine application when a large number of samples are to be treated (as here with 96×2 samples). The potential distortions to lake sediment DNA records due to taphonomic processes (production, transfer, preservation of DNA) that affect DNA in sediments are not fully known; we know however that:

- At sites with favourable DNA preservation conditions like in lake sediments, the DNA signal is proven to be reliable for several centuries. The signal can be preserved for several millennia if the preservation conditions are very good.[17]
- The first few years after deposition are critical for DNA preservation due to the biological activity at the sediment interface and the physical and chemical changes that occur in the uppermost sediment

layers.[18,19] Consequently, we chose to avoid the very recent deposits (for the sampling of modern periods) to overcome this issue; the top samples were sampled a few centimeters below the sediment surface (~ year 2000).

- Previous calibration studies on sedimentary DNA[20] have shown that some microbial eukaryotic groups are not well preserved in sediment even when the general conditions are favourable for DNA preservation, particularly for Cryptophyceae. In our study, a low amount of DNA reads were found for this group (<0.02% of DNA reads/sample, 4 OTUs in total); this group had a non significant contribution to micro-eukaryotic community changes (Supplementary Figure 7).

Furthermore, using our data, we explored if we could detect preferential degradation/modification of DNA for some groups of micro-eukaryotes; we found no evidence for differential DNA degradation between taxa relative richness (Supplementary Figure 3).

From this, we considered that the decay of DNA is probably marginal and a differential preservation of DNA between top and bottom is unlikely to have a significant effect on our results. Additionally, we considered different levels of taxonomy in order to circumvent the potential risk associated with the use of OTUs (artifactual increase of OTUs number, or loss of OTUs due to degradation/fragmentation). The choice of thresholds for the delineation of OTUs is critical, with potential risk of inflation of rare OTUs or, inversely, of lumping together OTUs with different distribution patterns. Universal thresholds also do not consider differences in substitution rates among lineages and may therefore not capture equivalent units of diversity.

The presence of active cells in sediments cannot be excluded; however, if we exclude restings cells considered as dormant but revivifiable,[21] and the taxa living at the surface of sediment (benthic protists in oxygenated zones), then, the rest of potentially active eukaryotic cells are most probably rare extremophile specialists representing a negligible fraction of the immense diversity of the total micro-eukaryote community. The micro-eukaryotic taxa we detected in this study are mainly known as planktonic groups; some (heterotrophs) that can live at the surface of oxygenated sediment were also found, but these taxa do not survive in hypoxic/anoxic conditions. From our data we could not identify any taxa known to live in deep sediments.

Sampling, processing and dating of sediment cores

Immediately after coring, the sediment-water interface was stabilized using floral foam. Cores were capped and tapped for transport and subsequent sampling in the lab. Each core was cut in the laboratory into two halves, one for multi-proxies analysis, including DNA, and one for core-logging and dating, both being stored in a 4° C cold room.

The sampling strategy had to be designed to meet the study objectives, i.e. characterizing micro-eukaryotic

diversity both for the last decades (top samples) and for ante-Great Acceleration (bottom samples), but also to fit with the methodological requirements of multi-proxies analyses in terms of sample volume and mass. The best balance was to work on samples covering at least 10-15 years. We combined chronological information from previous studies (when available) with chronostratigraphic marker such as atmospheric pollutant (lead), short-lived radionuclides ($^{137}Cs/^{210}Pb$), and radiocarbon dating. Geochemical analyses, including lead and other major and trace elements, were performed by X-ray fluorescence (AVAATECH XrF Core Scanner, Edytem Laboratory) at a 5 mm sampling step. Radionuclides ^{137}Cs and ^{210}Pb were measured by gamma spectrometry (Chrono-Environnement Laboratory). Radiocarbon measurements were performed using accelerator mass spectrometry in Poznan Radiocarbon Laboratory, Poland.

The main challenge in dating lacustrine sediments from the past two centuries is to estimate ages at the onset to the mid-19th century. Radiocarbon dating usually has multi-decadal uncertainties that are amplified by the presence of a plateau between 1600 and 1900 CE in the calibration curve. Using the ^{210}Pb chronometer is a robust alternative and/or complement but the mid-19th century is at the bottom limit of this dating method range (ca 150 years) and with greater uncertainties than for sediments for the last two or three decades. Lead (Pb) stratigraphic profiles were used to synchronize the lake sediment records with historical lead atmospheric deposition in the study area which was more particularly characterized by the increase in Pb fallout from the mid-1800s to the beginning of the 20th century following the industrial revolution and by the decrease in Pb deposition at the end of the 1970s due to the fall of the use of lead additives in European countries.[22,23] Eventually, chronostratigraphic markers such as lead can be differentially recorded in lakes, depending on the local human impact history (mining) sedimentation rate, and sediment geochemical properties. For all these reasons, it can be necessary to combine different methods, or to choose a specific one according to lake type.

Supplementary Table 2 summarizes how the depth and thickness of top/bottom sediment layers were set. For top samples, starting depth is always the stratigraphic zero of each core, i.e. the sediment-water interface. Then the thickness was defined as follows:

1. Using the depth of lead decrease from XrF core logging alone.
2. Same as 1 with addition of the depth of 1986 and/or 1963 ^{137}Cs atmospheric fallout (Chernobyl accident and maximum of atmospheric nuclear weapon tests, respectively). In this case the end of the 1970s lead peak is constrained between the two ^{137}Cs peaks.
3. Using radionuclides ($^{137}Cs/^{210}Pb$) based age-depth model. The difference with 2 is that ^{210}Pb chronometer enables to calculate an age for each analysed sediment samples over the last 150 yrs.
4. Using sedimentation rate calculated from ^{14}C ages.

5. For high-elevation lakes with low sedimentation rates and without chronostratigraphic marker visible along the upper part of the core, the thickness was set to 1.5 to 2 cm.

For bottom samples, depths and thickness were defined as follows:

1. Using the depth of the onset of lead increase from XrF core logging alone.
2. As with 1, with addition of radionuclides based (^{137}Cs/^{210}Pb) age-depth model
3. Using sedimentation rate calculated from ^{14}C ages.
4. For two Pyrenean high-elevation lakes with no chronological information, we had to set the depth of thickness of the bottom sediment layers according to the mean sedimentation rate in other lakes of this sub-region in the dataset, that were more precisely dated.

In any case, the selected bottom sediments are anterior in age to the Great Acceleration.

Subsampling for biomarker analyses and precautions for sample handling

Subsamplings were performed in a room isolated from DNA labs and equipped with a sterile airflow working station, after decontamination of work-surfaces. Sediment cores were split into two halves longitudinally. The material (cutting blades, splitting wires) was previously flamed with alcohol to remove potential DNA contaminants. The top surface of the core was scraped off perpendicular to the core pipe using sterile scalpels. Subsampling was performed from the bottom to the top of the core. To limit extraneous contamination, only the centre of each layer was used for molecular analysis, the subsamples were collected by transferring a small amount of sediment into a sterile tube using clean disposable spatulas (using a different sampling tool for each strata). These subsamples were stored at -20°C until DNA extraction. For replication purposes we constituted duplicate subsamples for each strata. Negative controls were included during subsampling procedures. For each subsample, DNA was quantified using a Quant-it PicoGreen kit (Invitrogen, Carlsbad, CA,USA) and was expressed in ng.g^{-1} of dry sediment. However, DNA quantities preserved in sediments should be interpreted cautiously because we cannot exclude that a small portion of the DNA concentration differences between top and bottom could be attributable to post-depositional degradation.

Photosynthetic pigments and organic carbon analyses

Organic carbon contents of sediment samples were analyzed using a vario MAX CNS analyzer (Elementar). Prior to analysis, the sediment samples were heat dried at 60°C during 24 h and carbonates were removed by the addition of 1 M HCl solution until effervescence ceased. Samples for photosynthetic pigment analysis were preserved in a cold room (4°C) prior to analysis. A subsample of ca. 1 g wet sediment was weighed and extracted overnight with 10 ml of acetone/water (90:10). Mechanical disruption and ultrasound were

not used to quantify the total carotenoids via spectrophotometer at the wavelengths of 450, 665 and 750 nm in spectrophotometer following recommendations by Guilizzoni et al.[24] Water content of sediment (after heating at 60°C) were measured in order to express the Total Carotenoïd (TC) concentrations, calculated following Amann et al,[25] in dry mass (TC in µg.dry g^{-1}).

Preparation of amplicons

Each PCR was performed in duplicate in a total volume of 25 µl containing 3 µl of 10X NH_4 reaction buffer, 1.2 µl of 50 mM $MgCl_2$, 0.25 µl of BioTaq (Bioline), 0.24 µl of 10 mM dNTP, 0.36 µl of 0.5 µg.$µl^{-1}$ BSA and 1 µl of each primer. The amplification conditions included initial denaturation at 94°C for 10 min followed by 35 cycles of 1 min at 94°C, 1 min at 55°C and 1 min 30 s at 72°C. The amplicons were then subjected to a final 10 min extension at 72°C. The two PCR products obtained for a given DNA extraction were pooled and purified using Illustra™ GFX™ PCR DNA and Gel Band Purification Kit (GE Healthcare Life Sciences, Velizy Villacoublay, France), and quantified using a Quant-it PicoGreen kit (Invitrogen, Carlsbad, CA, USA) on a Fluoroskan Ascent™ FL (ThermoLabsystems, Beverly, MA, USA).

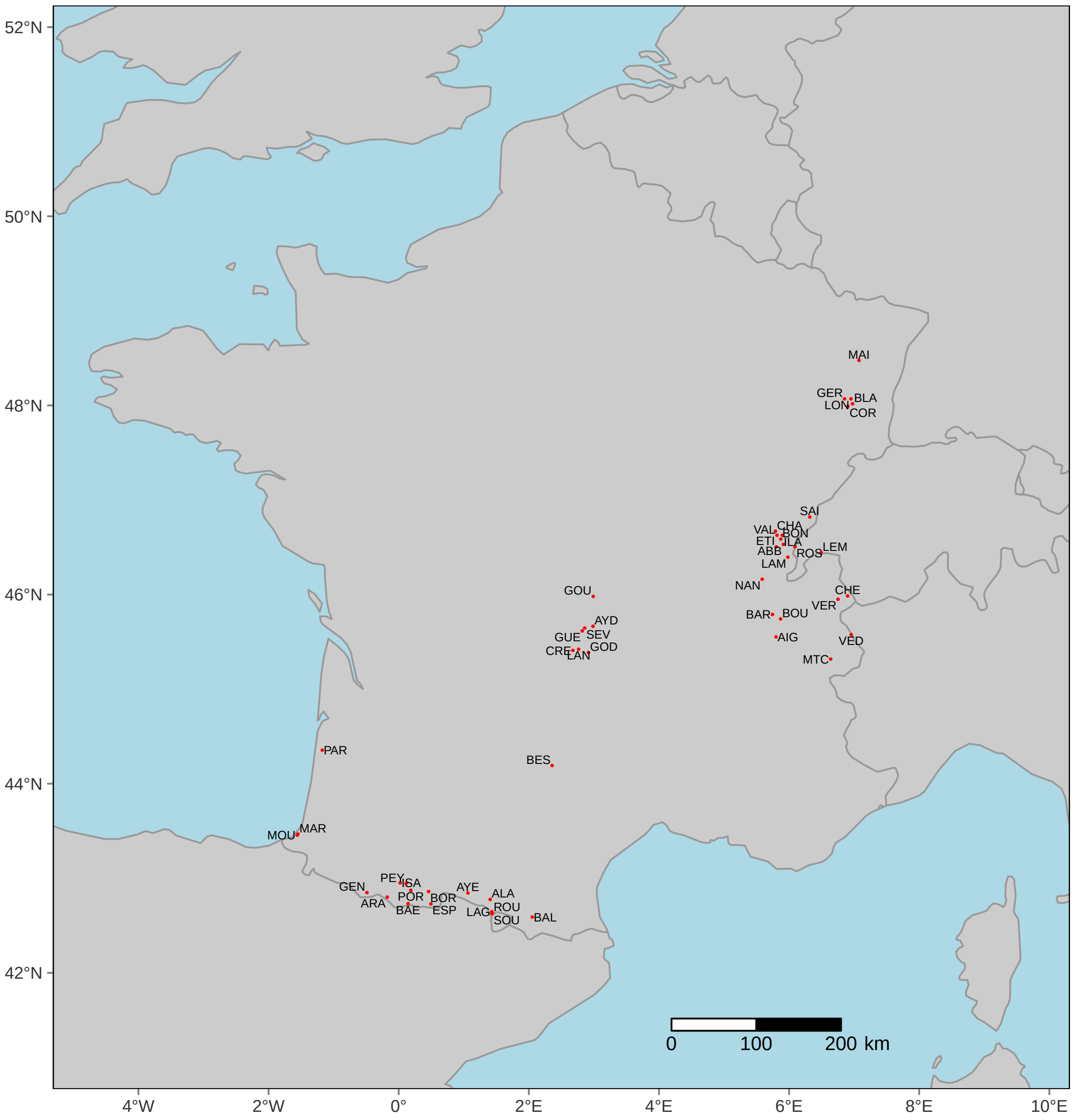

Supplementary Figure 1. Map of the 48 lakes included in the study. Lake names corresponding to three-letter codes can be found in Supplementary Table 1.

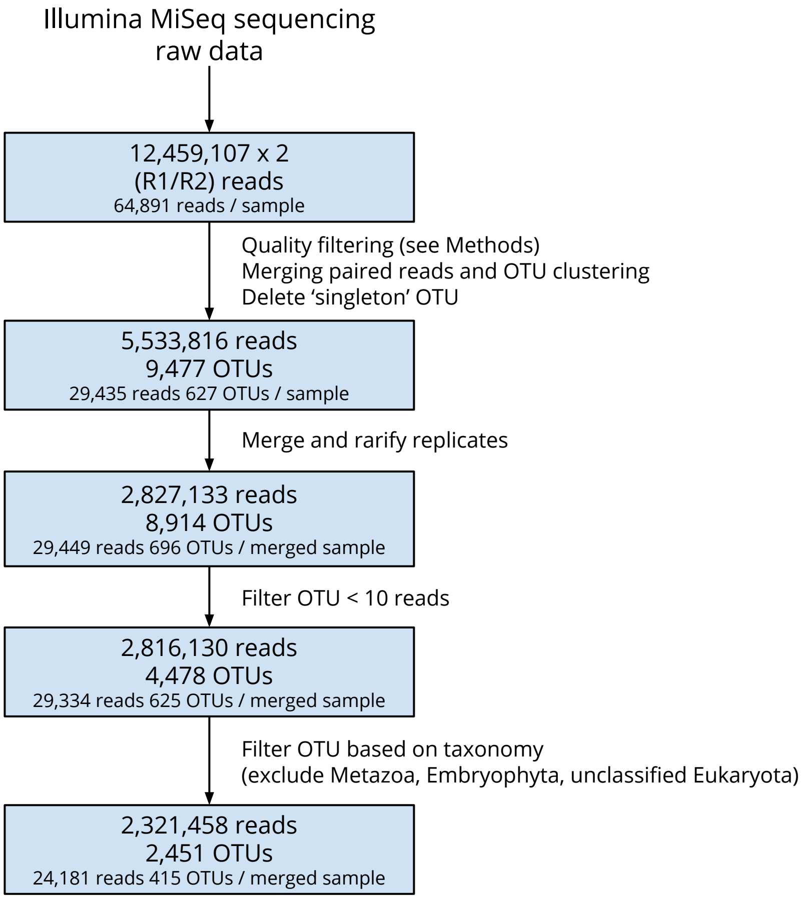

Supplementary Figure 2. Tracking of the number of DNA reads and OTUs during the filtering and data pre-processing steps. Numbers written in small print indicate the mean values per sample (or merged sample after merging).

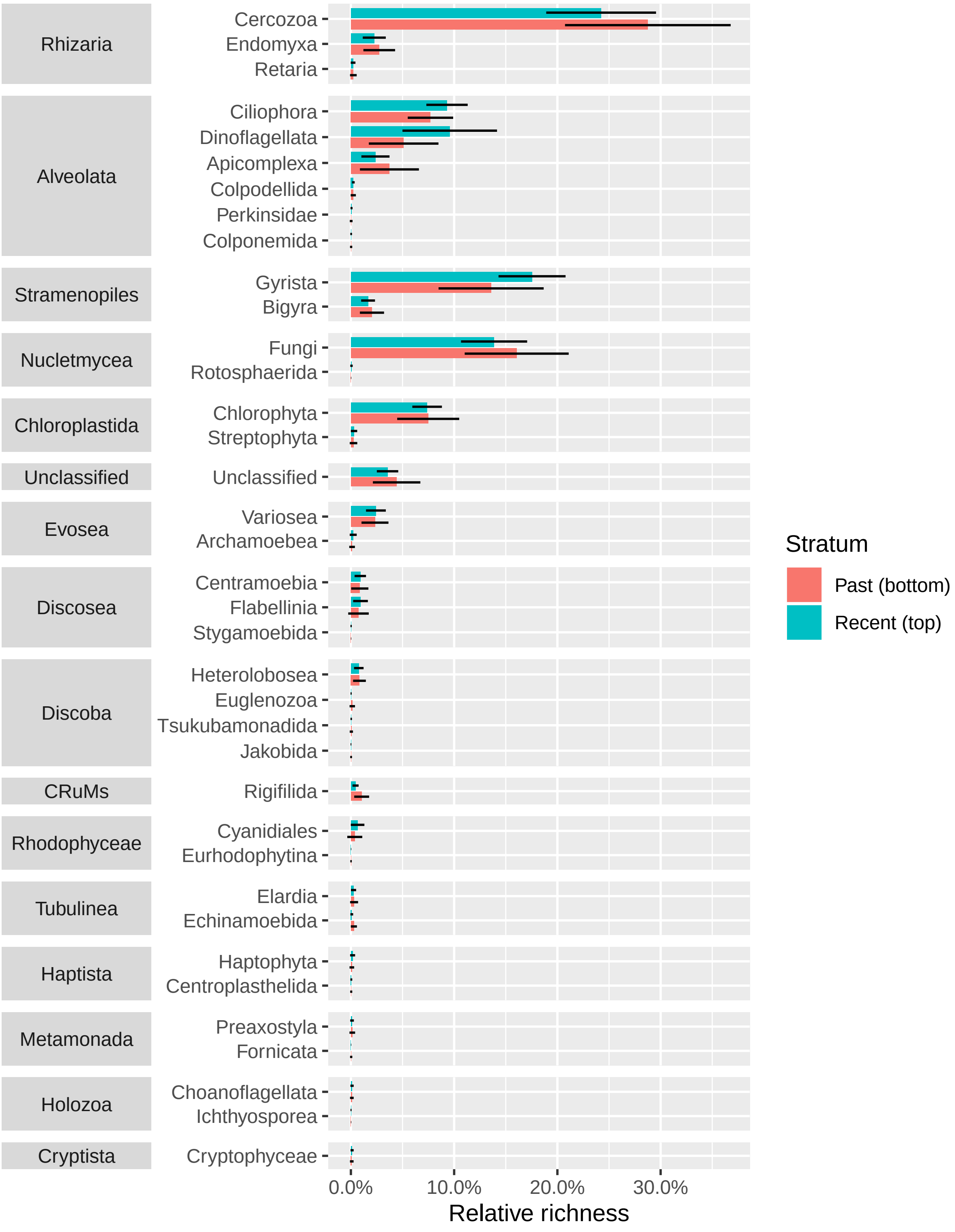

Supplementary Figure 3. Mean relative richness (number of distinct OTUs) for each taxa computed for the bottom and the top rarefied samples across all lakes (n = 48). Horizontal black lines show the standard error.

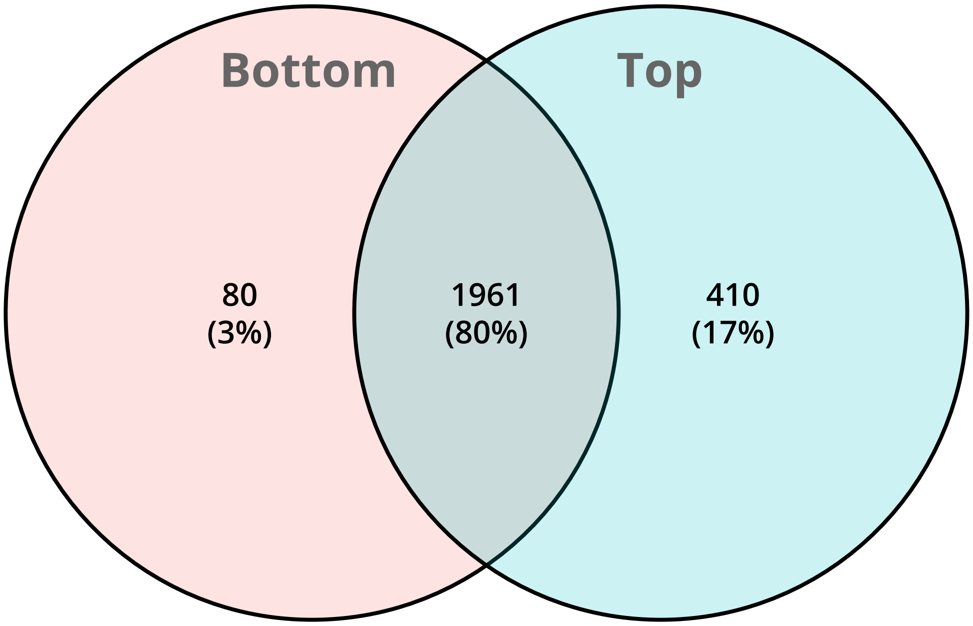

Supplementary Figure 4. Venn diagram showing the number and proportion of OTUs specific to the bottom and top strata, or common to both strata (intersection).

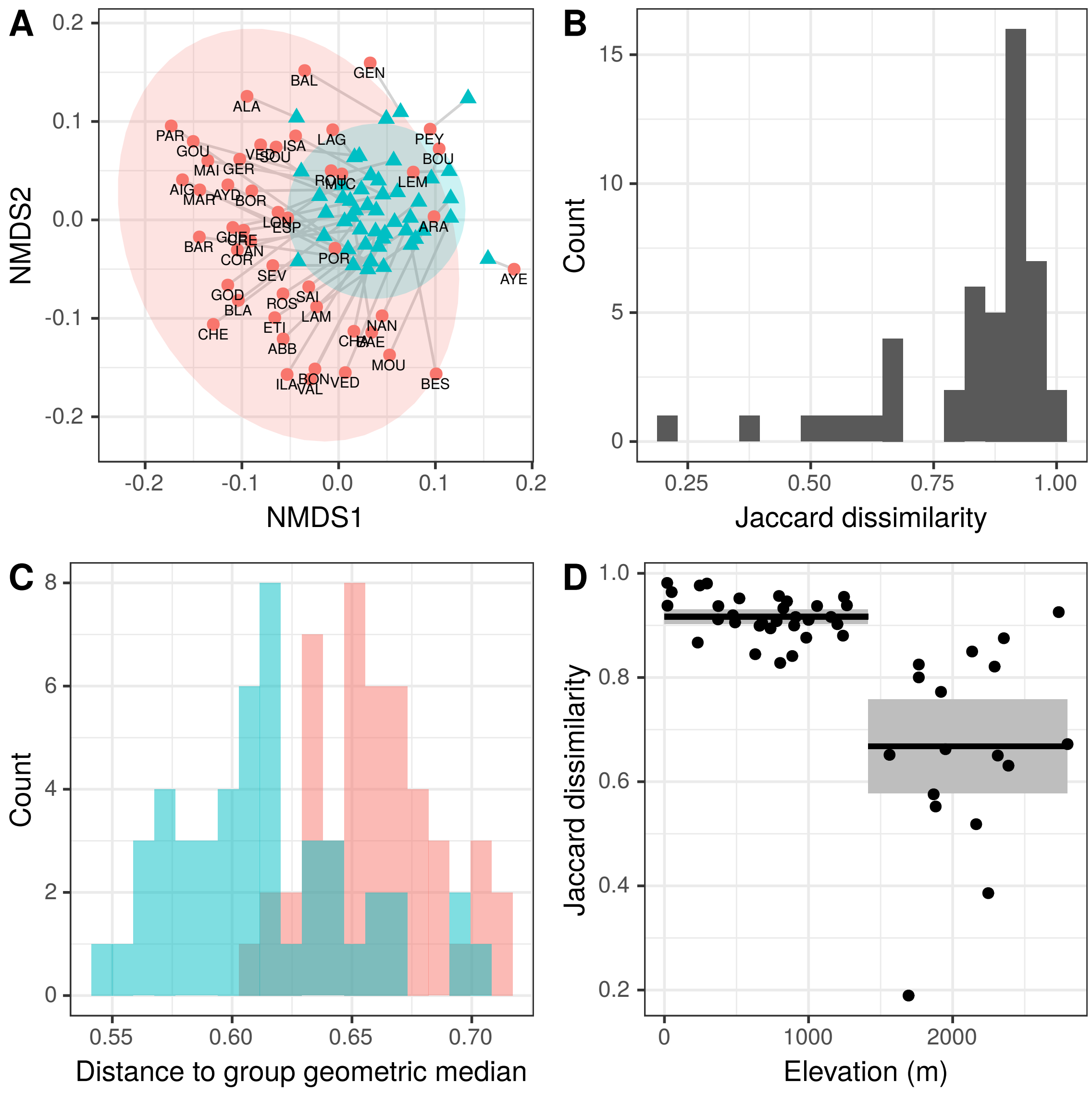

Supplementary Figure 5. Community analyses of the OTUs table reproduced with the Jaccard dissimilarity index. (*A*) NMDS of community compositions of the recent (blue triangles) and past (red circles) samples with 95% confidence ellipses represented for each group. Grey lines connect recent and past samples from the same lake. Stress = 0.24. (*B*) Distribution of Jaccard index values computed for each lake between recent and past samples. (*C*) Distribution of the distances between samples and group geometric median for recent (blue) and past (red) samples. (*D*) Relationship between lake elevation and community dissimilarity (Jaccard index) between the recent and past strata (n = 48 lakes). Fitted regression tree model is represented by black lines (mean values). Grey shading represents the 95% confidence intervals around means.

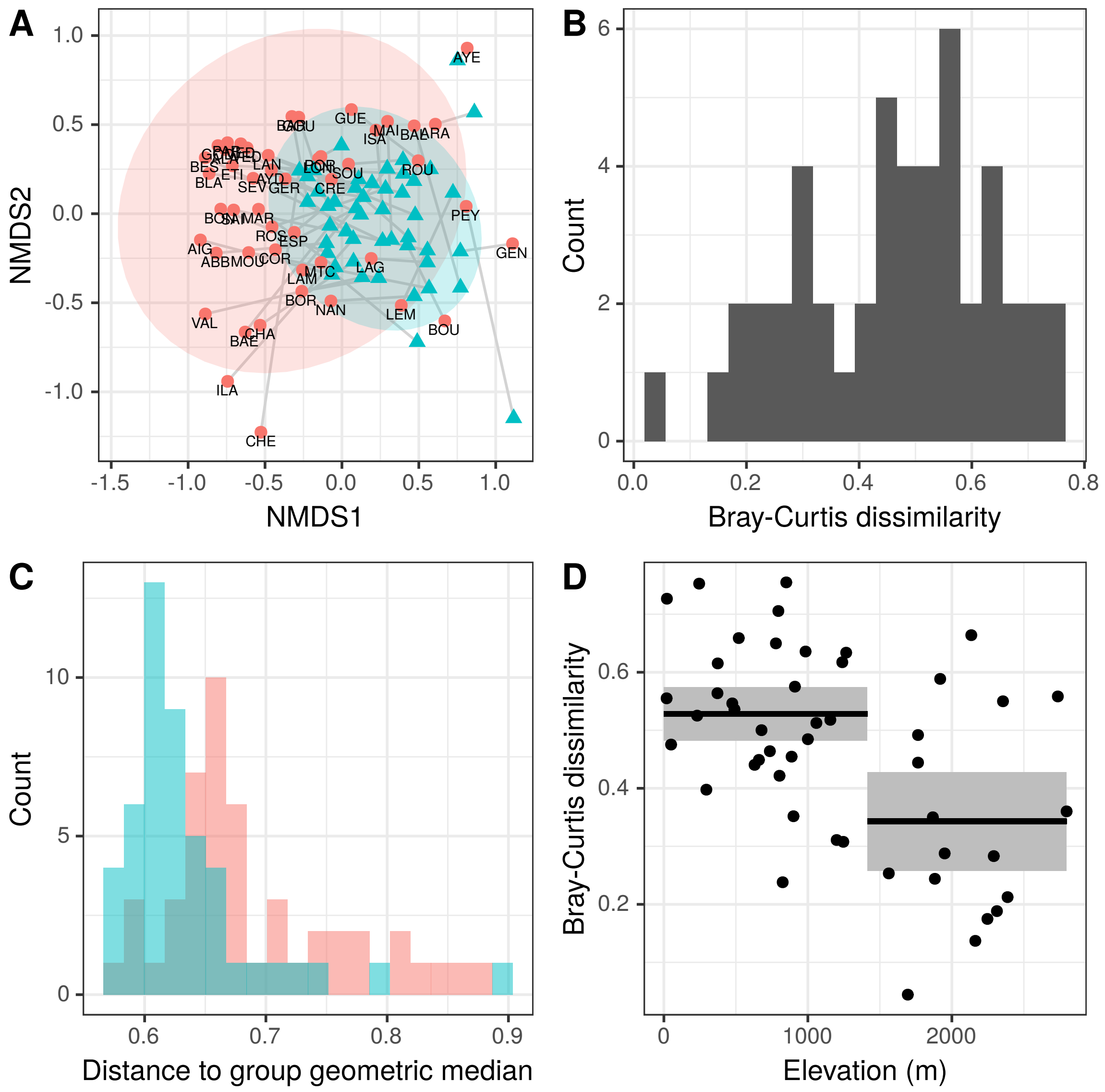

Supplementary Figure 6. Community analyses reproduced with the data aggregated at the 2nd taxonomic rank of Adl et al.[26] (*A*) NMDS of community compositions of the recent (blue triangles) and past (red circles) samples with 95% confidence ellipses represented for each group. Grey lines connect recent and past samples taken within the same lake. Stress = 0.19. (*B*) Distribution of Bray-Curtis index values computed for each lake between recent and past samples. (*C*) Distribution of the distances between samples and group geometric median for recent (blue) and past (red) samples. (*D*) Relationship between lake elevation and community dissimilarity (Bray-Curtis index) between the recent and past strata (n = 48 lakes). Fitted regression tree model is represented by black lines (mean values). Grey shading represents the 95% confidence intervals around means.

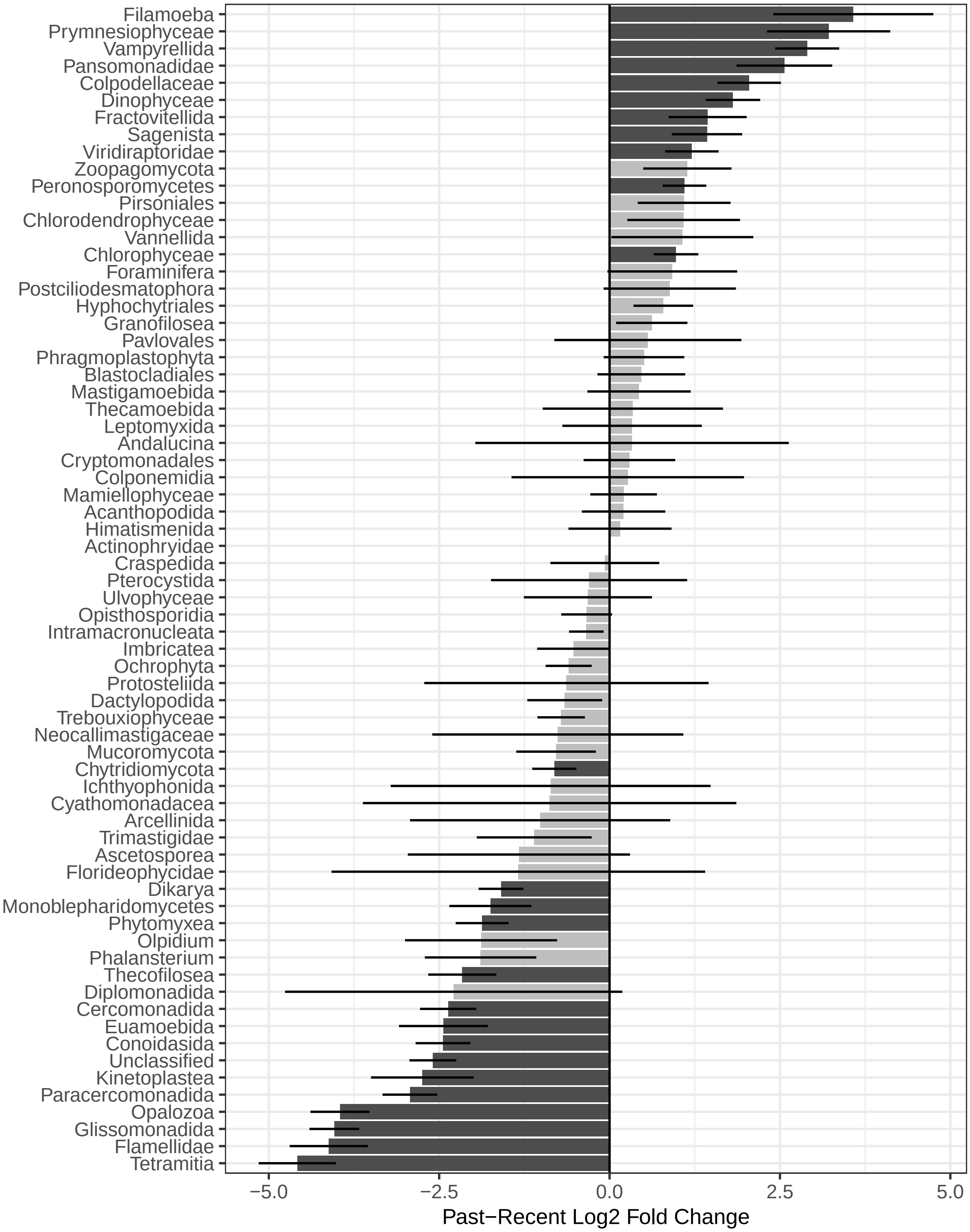

Supplementary Figure 7. Magnitude of change (expressed in log2 fold change) in data aggregated at the 3rd taxonomy rank of Adl et al[26] between the past and recent strata (n = 48 lakes), as estimated by the DESeq2 analysis.[26] Dark bars represent groups for which the change was found significant (two-sided Wald test corrected with the Benjamini & Hochberg method p-value < 0.05). Horizontal lines show the standard error. The detailed results of the analysis are given in Supplementary Table 5.

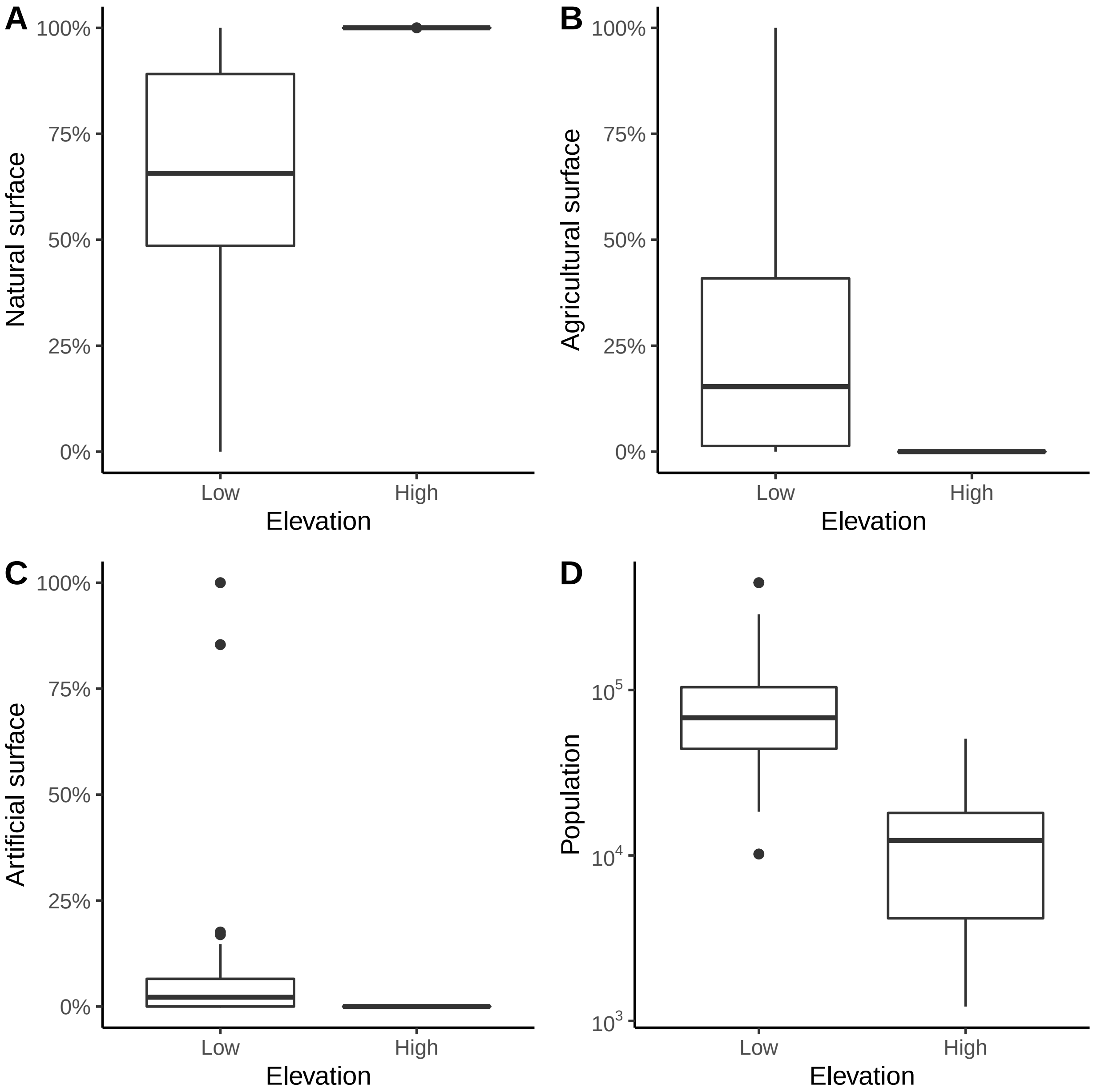

Supplementary Figure 8. Proportion of (*A*) natural, (*B*) agricultural and (*C*) artificial surfaces in watersheds of high- ($>$ 1400 m) and low-elevation ($\leq$ 1400 m) lakes. The landcover analysis was conducted on a subset of 39 lakes for which data were available (10 high-elevation lakes and 29 low-elevation lakes). (*D*) Total human population living within a radius of 20 km of high-elevation lakes (n = 17) and low-elevation lakes (n = 31). Boxplots depict medians, first and third quartiles, and full ranges (bounded at 1.5 $\times$ interquartile range).

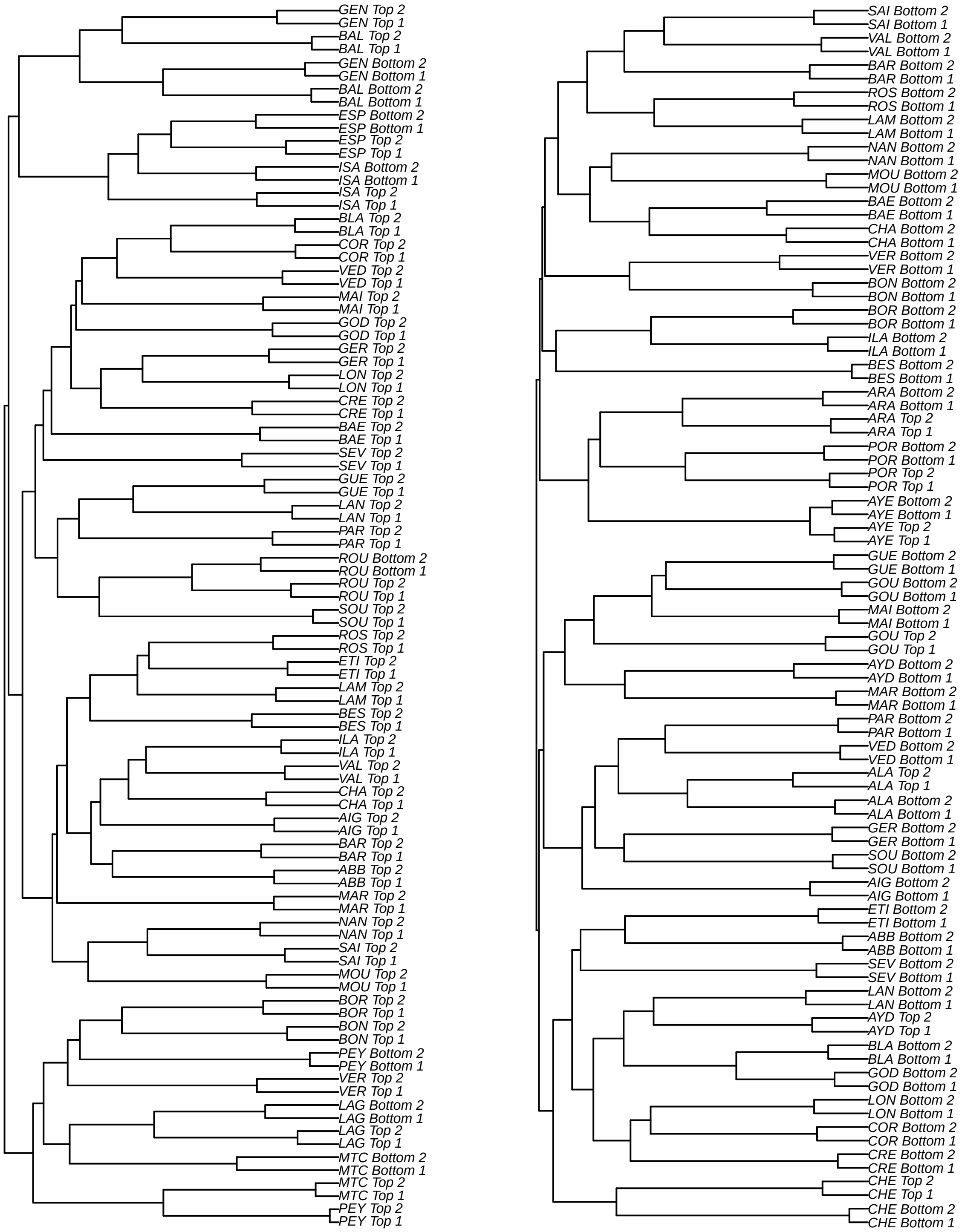

Supplementary Figure 9. Hierarchical clustering of the OTU matrix (Bray Curtis dissimilarity) including the technical replicates. The code of each sample corresponds to the combination of the lake (three-letter code; see Supplementary Table 1), stratum (top or bottom) and replicate number (1 or 2). Lakes Bourget and Léman are not represented as they have only one replicate. For better readability, the tree has been divided in two parts.

Code	Name	Longitude	Latitude	Elevation	Depth (max)	Trophic status
PAR	Parentis	-1.174	44.355	19	20	Eutrophic
MOU	Mouriscot	-1.558	43.456	21	10	Eutrophic
MAR	Marion	-1.548	43.468	50	23	Eutrophic
BOU	Bourget	5.869	45.742	231	147	Oligo-mesotrophic
BES	Besse	2.359	44.192	245	10	Unknown
BAR	Barterand	5.744	45.790	295	15	Mesotrophic
LEM	Léman	6.492	46.444	372	309	Mesotrophic
AIG	Aiguebelette	5.800	45.553	374	71	Oligo-mesotrophic
NAN	Nantua	5.587	46.162	475	43	Mesotrophic
CHA	Chalain	5.790	46.671	490	32	Mesotrophic
VAL	Val	5.815	46.627	520	25	Mesotrophic
GOU	Tazenat	2.990	45.980	630	66	Oligo-mesotrophic
GER	Gérardmer	6.852	48.070	660	38	Mesotrophic
MAI	Maix	7.075	48.476	678	15	Unknown
LON	Longemer	6.951	48.070	736	34	Mesotrophic
ILA	Ilay	5.898	46.627	778	32	Meso-eutrophic
ETI	Etival	5.804	46.504	795	10	Mesotrophic
BON	Bonlieu	5.873	46.587	803	14	Mesotrophic
AYD	Aydat	2.986	45.664	825	15	Eutrophic
SAI	Saint-Point	6.318	46.820	850	42	Meso-eutrophic
COR	Corbeaux	6.904	47.991	887	27	Unknown
CRE	Crégut	2.677	45.409	900	26	Eutrophic
ABB	Abbaye	5.912	46.531	910	19.5	Mesotrophic
BLA	Blanchemer	6.977	48.018	984	15	Unknown
LAN	Landie	2.766	45.422	1000	21	Meso-eutrophic
ROS	Rousses	6.089	46.505	1059	18	Oligo-mesotrophic
LAM	Lamoura	5.981	46.395	1156	9	Unknown
SEV	Serviere	2.859	45.646	1200	29	Mesotrophic
GOD	Godivelle	2.917	45.387	1239	44	Oligotrophic
GUE	Guéry	2.822	45.616	1246	20	Unknown
VER	Vert	6.753	45.950	1266	9	Unknown
ISA	Isaby	0.023	42.951	1562	6	Oligotrophic
AYE	Ayes	1.064	42.844	1694	10	Oligotrophic
BOR	Bordères	0.461	42.860	1765	18	Oligotrophic
BAL	Balcère	2.053	42.590	1765	14	Mesotrophic
ALA	Alate	1.406	42.776	1868	10	Ultra-oligotrophic
ESP	Espingo	0.495	42.729	1882	8	Oligotrophic
PEY	Peyrelade	0.114	42.943	1919	28	Oligotrophic
GEN	Gentau	-0.488	42.848	1950	20	Mesotrophic
CHE	Chéserys	6.900	45.985	2135	6	Oligotrophic
ROU	Roumazet	1.435	42.634	2163	10	Oligotrophic
ARA	Arratille	-0.175	42.801	2247	12	Oligotrophic
SOU	Soucarrane	1.434	42.626	2291	10	Oligotrophic
POR	Port Bielh	0.187	42.873	2313	19	Oligotrophic
BAE	Barroude	0.144	42.731	2355	9	Oligotrophic
LAG	Gardelle	1.432	42.647	2387	27	Oligotrophic
VED	Verdet	6.955	45.576	2736	12	Oligotrophic
MTC	Mont Coua	6.639	45.318	2797	10	Oligotrophic
**Not included for DNA analyses**						
AUL	Aulnes	4.790	43.593	11	<10	Unknown
CAZ	Cazaux	1.163	44.487	12	22.3	Meso-eutrophic
ENT	Entressens	4.907	43.604	36	8	Eutrophic
MON	Montcineyre	2.896	45.460	1180	22	Oligo-mesotrophic
BLC	Blanc	6.966	45.580	2850	40	Oligotrophic

Supplementary Table 1. List of the lakes included in the study with their three-letter code, geographic coordinates (decimal degrees; WGS84), elevation (m) and maximum depth (m). The trophic status, when referenced, is based on 3 parameters used in the Water Framework Directive, i.e. Secchi disk value, phosphorus concentration in the water and chlorophyll *a* concentration in the euphotic zone.

Name	Coring year	XrF data	^{14}C	Radionuclides	1986 ^{137}Cs fallout	1963 ^{137}Cs fallout	Onset of lead decrease (XrF)	Increase in lead (XrF)	Depth Top	Depth Bottom	Method Top/Bottom
Parentis	2015	x					21	70	0-6	62-66	1/1
Mouriscot	2015	x					12	45	0-4	42-44	1/1
Marion	2015	x		^{137}Cs		49-56	49	80	0-14	80-85	2/1
Bourget[a]	2010			^{137}Cs, ^{210}Pb					0-2		3/2
Besse	2015	x					25.5	72	0-6	68-72	1/1
Barterand	2015	x					7.5	33	0-2	30-32	1/1
Léman[b]	2010			^{137}Cs, ^{210}Pb					0-2		3/2
Aiguebelette	2015	x					6	21	0-2	19-21	1/1
Nantua	2015	x		^{137}Cs, ^{210}Pb	9-10	9-20	15.5	90	0-5	84-89	3/2
Chalain	2015	x		^{137}Cs	5.5-6	11-12	8.5	20	0-4	16-18.5	2/1
Val	2015	x					5	23	0-3	20-23	1/1
Tazenat	2015	x					5	26	0-2.5	23.5-25.5	1/1
Gérardmer[c]	2013	x	x	^{137}Cs, ^{210}Pb	4-5	7-8	7	16	0-3	12-14	3/2
Maix	2015	x		^{137}Cs	6-8	20-22	13	62	0-4	50-55	2/1
Longemer[c]	2013	x	x	^{137}Cs, ^{210}Pb	7-8	10-11	9.5	22	0-4	16-18	3/2
Ilay	2015	x					4	37	0-3	30-34	1/1
Etival	2015	x					9	40	0-4	36-40	1/1
Bonlieu	2013		x	^{137}Cs	4-6	8-10	NA	24	0-4	22-24	2/1
Aydat[d]	2015	x	x	^{137}Cs, ^{210}Pb			33	80	0-10	65-70	1/1
Saint-Point[e]	2015	x		^{137}Cs, ^{210}Pb			7.5	22	0-2	20-21	1/1
Corbeaux	2015	x	x	^{137}Cs	5-6	9-10	8.5	20	0-4	18-20	2/1
Crégut[f]	2015	x					21	42	0-5	40-42	1/1
Abbaye	2015	x		^{137}Cs	10-12		18	50	0-5	45-47.5	2/1
Blanchemer	2015	x		^{137}Cs	12-14		16	48	0-3	44-48	2/1
Landie	2015	x		^{137}Cs, ^{210}Pb	NC	27-30	26	56	0-9	47-50	3/2
Rousses	2015	x					9.5	30	0-5	26-30	1/1
Lamoura	2015	x					24	37	0-10	32-37	1/1
Serviere	2015	x					8	22	0-3.5	20-22	1/1
Godivelle	2015	x		^{137}Cs, ^{210}Pb	1-1.5	NC	3.5	18	0-2	16-18	3/2
Guéry	2015	x		^{137}Cs	12-14	20-22	16	74	0-5	71-74	2/1
Vert	2015	x					2	8	0-2	6-8	1/1
Isaby	2014		x						0-1	5-6	4/3
Ayes	2015								0-1.5	5-6.5	5/4
Bordères	2016	x	x				2	10	0-2	5-6.5	1/1
Balcère	2013								0-2	5-6.5	5/4
Alate	2016	x					NC	10	0-1.5	5-6.5	5/1
Espingo	2016	x	x				NC	6.5	0-2	5-6.5	4/3
Peyrelade	2016	x	x				2.5	9	0-1.5	5-6.5	1/3
Gentau	2014		x						0-1.5	4-5.5	4/3
Chéserys	2013	x	x				NC	3.5	0-0.5	2-2.5	4/3
Roumazet	2016	x	x				NC	9	0-1.5	5-6.5	5/3
Arratille	2013		x						0-1.5	6-7.5	4/3
Soucarrane	2016	x					NC	7	0-1.5	5-6.5	4/3
Port Bielh	2014		x						0-1.5	2.5-4	4/3
Barroude	2013		x						0-1.5	6-7.5	4/3
Lagardelle	2016	x	x				NC	NC	0-1.5	5-6.5	4/3
Verdet	2015	x		^{137}Cs, ^{210}Pb	NC	7-7.5	NC	NC	0-2	19-21	3/2
Mont Coua	2015	x		^{137}Cs, ^{210}Pb	NC	NC	NC	9	0-1	8-9	3/2

[a] Additional source: Giguet-Covex et al[27] ;
[b] Additional source: Alric et al[28] ;
[c] Additional source: Belle et al[29] ;
[d] Previous studies of Sarazin et al[30] and Lavrieux et al[31] indicated a mean sedimentation rate of 0.46 and 0.52 over the last century. Furthermore, two floods events dated at 1907 and 1846 by Lavrieux et al[31] has been identified along the 2015 core from XrF logging at 57.5 and 75.5 cm, respectively.
[e] Nedjai et al[32] indicated a mean sedimentation rate of 0.21 $cm.yr^{-1}$ over the last century.
[f] A strong change in terrigenous elements (e.g. Ti) that occured at 31 cm along the 2015 core corresponds to the year 1970 when the watershed surface was artificially increased from 1.5 to 86 km^2.

Supplementary Table 2. Data supporting the dating and sampling of sediment cores. For each lakes is reported: the year of coring, the availability of Xrf, ^{14}C and radionuclides data, the depth (cm) for 1986 and 1963 ^{137}Cs fallouts and lead increase/decrease, the depth (cm) for top and bottom samples. NC stands for “not clear”. Methods used to determine the depth of top and bottom samples are indicated in the last column where numbers refer to methods described in Supplementary Methods.

	Degrees of freedom	Sum of squares	F-stat	Partial R2	P-value
Top-Bottom	1	2.522	7.057	0.07	<0.001
Residuals	94	33.588		0.93	
Total	95	36.109		1.00	

Supplementary Table 3. Detailed table of the PERMANOVA. The p-value is computed using a two-sided permutational test of the pseudo F-statistic.

Trophic Group	Base Mean	log2 Fold Change	Stat	Padj
Consumers	29783.064	-3.248	-11.575	0.000
Mixotrophs	1630.349	1.395	4.201	0.000
Parasites	1358.376	-0.979	-4.396	0.000
Photosynthetics	3273.439	0.670	2.357	0.022
Saprotrophs	929.196	-0.930	-2.730	0.009
Unclassified	1703.652	0.246	1.120	0.263

Supplementary Table 4. Detailed results of the DESeq2 analysis conducted on data aggregated by trophic groups. For each group, column *Base Mean* indicates the average of the normalized count values, dividing by size factors, *log2 Fold Change* is the magnitude of change between the past and recent strata, column *Stat* is the Wald statistic and *Padj* the p-value of the test corrected with the Benjamini & Hochberg method.

Taxonomic rank (Adl et al. 2019)						
Rank 1	Rank 2	Rank 3	Base Mean	log2 Fold Change	Stat	Padj
Alveolata	Apicomplexa	Conoidasida	181.992	-2.445	-6.083	0.000
	Ciliophora	Intramacronucleata	1631.737	-0.340	-1.340	0.348
		Postciliodesmatophora	0.858	0.884	0.910	0.555
	Colpodellida	Colpodellaceae	27.510	2.049	4.398	0.000
	Colponemida	Colponemidia	0.313	0.267	0.156	0.913
	Dinoflagellata	Dinophyceae	2044.408	1.811	4.536	0.000
Chloroplastida	Chlorophyta	Chlorodendrophyceae	4.650	1.087	1.314	0.348
		Chlorophyceae	806.737	0.974	2.970	0.009
		Mamiellophyceae	3.879	0.206	0.421	0.843
		Trebouxiophyceae	49.057	-0.710	-2.039	0.101
		Ulvophyceae	0.190	-0.318	-0.338	0.848
	Streptophyta	Phragmoplastophyta	6.755	0.505	0.854	0.581
Cryptista	Cryptophyceae	Cryptomonadales	1.714	0.291	0.433	0.843
		Cyathomonadacea	0.109	-0.880	-0.321	0.848
Discoba	Euglenozoa	Kinetoplastea	0.841	-2.748	-3.649	0.001
	Heterolobosea	Tetramitia	900.089	-4.581	-8.074	0.000
	Jakobida	Andalucina	0.247	0.329	0.143	0.913
Discosea	Centramoebia	Acanthopodida	6.453	0.205	0.335	0.848
		Himatismenida	7.888	0.153	0.203	0.892
	Flabellinia	Dactylopodida	33.011	-0.658	-1.198	0.413
		Thecamoebida	0.337	0.341	0.258	0.873
		Vannellida	0.986	1.068	1.025	0.519
Evosea	Archamoebea	Mastigamoebida	4.363	0.432	0.570	0.790
	Variosea	Filamoeba	1.652	3.575	3.043	0.008
		Flamellidae	1456.930	-4.120	-7.178	0.000
		Fractovitellida	204.109	1.440	2.517	0.032
		Phalansterium	0.675	-1.892	-2.315	0.052
		Protosteliida	0.105	-0.633	-0.303	0.849
Haptista	Centroplasthelida	Pterocystida	0.153	-0.301	-0.209	0.892
	Haptophyta	Pavlovales	0.488	0.562	0.409	0.843
		Prymnesiophyceae	2.830	3.215	3.557	0.001
Holozoa	Choanoflagellata	Craspedida	1.736	-0.070	-0.087	0.944
	Ichthyosporea	Ichthyophonida	0.046	-0.864	-0.368	0.848
Metamonada	Fornicata	Diplomonadida	0.277	-2.288	-0.924	0.555
	Preaxostyla	Trimastigidae	1.699	-1.106	-1.312	0.348
Nucletmycea	Fungi	Blastocladiales	8.146	0.465	0.723	0.666
		Chytridiomycota	988.920	-0.812	-2.506	0.032
		Dikarya	148.872	-1.593	-4.835	0.000
		Monoblepharidomycetes	3.690	-1.748	-2.900	0.011
		Mucoromycota	199.711	-0.785	-1.344	0.348
		Neocallimastigaceae	0.144	-0.760	-0.412	0.843
		Olpidium	0.381	-1.885	-1.690	0.194
		Opisthosporidia	112.954	-0.337	-0.902	0.555
		Zoopagomycota	16.687	1.141	1.761	0.172
Rhizaria	Cercozoa	Cercomonadida	1243.487	-2.369	-5.739	0.000
		Glissomonadida	3890.922	-4.039	-11.058	0.000
		Granofilosea	90.695	0.619	1.183	0.413
		Imbricatea	55.735	-0.531	-0.998	0.528
		Pansomonadidae	21.365	2.565	3.652	0.001

(continued)

Taxonomic rank (Adl et al. 2019)						
Rank 1	Rank 2	Rank 3	Base Mean	log2 Fold Change	Stat	Padj
		Paracercomonadida	377.560	-2.929	-7.284	0.000
		Thecofilosea	3450.787	-2.162	-4.324	0.000
		Viridiraptoridae	16.026	1.207	3.078	0.007
	Endomyxa	Ascetosporea	0.122	-1.330	-0.816	0.600
		Phytomyxea	64.054	-1.870	-4.825	0.000
		Vampyrellida	75.070	2.900	6.184	0.000
	Retaria	Foraminifera	0.750	0.920	0.967	0.540
Rhodophyceae	Eurhodophytina	Florideophycidae	0.119	-1.339	-0.489	0.834
Stramenopiles	Bigyra	Opalozoa	482.961	-3.956	-9.119	0.000
		Sagenista	7.928	1.429	2.769	0.016
	Gyrista	Actinophryidae	0.000	0.000	0.000	1.000
		Hyphochytriales	2.588	0.789	1.800	0.168
		Ochrophyta	5250.107	-0.600	-1.777	0.171
		Peronosporomycetes	57.024	1.099	3.435	0.002
		Pirsoniales	19.962	1.094	1.605	0.224
Tubulinea	Elardia	Arcellinida	0.218	-1.019	-0.534	0.807
		Euamoebida	10.894	-2.438	-3.737	0.001
		Leptomyxida	0.506	0.330	0.323	0.848
Unclassified	Unclassified	Unclassified	4859.976	-2.594	-7.556	0.000

Supplementary Table 5. Detailed results of the DESeq2 analysis conducted on data aggregated at the 3rd taxonomy rank of Adl et al.[26] Ranks 1 and 2 are given for guidance. For each taxa, column *Base Mean* indicates the average of the normalized count values, dividing by size factors, *log2 Fold Change* is the magnitude of change between the past and recent strata, column *Stat* is the Wald statistic and *Padj* the p-value of the test corrected with the Benjamini & Hochberg method.

Supplementary References

1. Boere, A. C., Rijpstra, W. I. C., De Lange, G. J., Sinninghe Damsté, J. S. & Coolen, M. J. L. Preservation potential of ancient plankton DNA in pleistocene marine sediments: Sedimentary ancient DNA. *Geobiology* **9**, 377–393 (2011).

2. Rizzi, E., Lari, M., Gigli, E., De Bellis, G. & Caramelli, D. Ancient DNA studies: New perspectives on old samples. *Genet Sel Evol* **44**, 1–19 (2012).

3. Torti, A., Lever, M. A. & Jørgensen, B. B. Origin, dynamics, and implications of extracellular DNA pools in marine sediments. *Marine Genomics* **24**, 185–196 (2015).

4. Capo, E. *et al.* Tracking a century of changes in microbial eukaryotic diversity in lakes driven by nutrient enrichment and climate warming. *Environmental Microbiology* **19**, 2873–2892 (2017).

5. Lloyd, K. G., MacGregor, B. J. & Teske, A. Quantitative PCR methods for RNA and DNA in marine sediments: Maximizing yield while overcoming inhibition. *FEMS Microbiology Ecology* **72**, 143–151 (2010).

6. Turon, V., Trably, E., Fayet, A., Fouilland, E. & Steyer, J.-P. Raw dark fermentation effluent to support heterotrophic microalgae growth: Microalgae successfully outcompete bacteria for acetate. *Algal Research* **12**, 119–125 (2015).

7. Rimet, F. *et al.* The observatory on LAkes (OLA) database: Sixty years of environmental data accessible to the public. *Journal of Limnology* (2020) doi:10.4081/jlimnol.2020.1944.

8. Anderson-Carpenter, L. L. *et al.* Ancient DNA from lake sediments: Bridging the gap between paleoecology and genetics. *BMC Evolutionary Biology* **11**, 30 (2011).

9. Ficetola, G. F. *et al.* DNA from lake sediments reveals long-term ecosystem changes after a biological invasion. *Science Advances* **4**, eaar4292 (2018).

10. Sjögren, P. *et al.* Lake sedimentary DNA accurately records 20th century introductions of exotic conifers in scotland. *New Phytologist* **213**, 929–941 (2017).

11. Parducci, L. *et al.* Shotgun environmental DNA, pollen, and macrofossil analysis of lateglacial lake sediments from southern sweden. *Frontiers in Ecology and Evolution* **7**, 189 (2019).

12. Ogram, A., Sayler, G. S., Gustin, D. & Lewis, R. J. DNA adsorption to soils and sediments. *Environ. Sci. Technol.* **22**, 982–984 (1988).

13. Dell'Anno, A., Bompadre, S. & Danovaro, R. Quantification, base composition, and fate of extracellular

DNA in marine sediments. *Limnology and Oceanography* **47**, 899–905 (2002).

14. Pedersen, M. W. *et al.* Ancient and modern environmental DNA. *Philosophical Transactions of the Royal Society B: Biological Sciences* **370**, 20130383 (2015).

15. Domaizon, I., Winegardner, A., Capo, E., Gauthier, J. & Gregory-Eaves, I. DNA-based methods in paleolimnology: New opportunities for investigating long-term dynamics of lacustrine biodiversity. *J Paleolimnol* **58**, 1–21 (2017).

16. Giguet-Covex, C. *et al.* New insights on lake sediment DNA from the catchment: Importance of taphonomic and analytical issues on the record quality. *Sci Rep* **9**, 14676 (2019).

17. Epp, L. S. A global perspective for biodiversity history with ancient environmental DNA. *Molecular Ecology* **28**, 2456–2458 (2019).

18. Vuillemin, A. *et al.* Preservation and significance of extracellular DNA in ferruginous sediments from lake towuti, indonesia. *Frontiers in Microbiology* **8**, 1440 (2017).

19. Capo, E., Domaizon, I., Maier, D., Debroas, D. & Bigler, C. To what extent is the DNA of microbial eukaryotes modified during burying into lake sediments? A repeat-coring approach on annually laminated sediments. *J Paleolimnol* **58**, 479–495 (2017).

20. Capo, E., Debroas, D., Arnaud, F. & Domaizon, I. Is planktonic diversity well recorded in sedimentary DNA? Toward the reconstruction of past protistan diversity. *Microb Ecol* **70**, 865–875 (2015).

21. Ellegaard, M. *et al.* Dead or alive: Sediment DNA archives as tools for tracking aquatic evolution and adaptation. *Communications Biology* **3**, 1–11 (2020).

22. Renberg, I., Bindler, R. & Brännvall, M.-L. Using the historical atmospheric lead-deposition record as a chronological marker in sediment deposits in europe. *The Holocene* **11**, 511–516 (2001).

23. Schwikowski, M. *et al.* Post-17th-century changes of european lead emissions recorded in high-altitude alpine snow and ice. *Environ. Sci. Technol.* **38**, 957–964 (2004).

24. Guilizzoni, P., Marchetto, A., Lami, A., Gerli, S. & Musazzi, S. Use of sedimentary pigments to infer past phosphorus concentration in lakes. *J Paleolimnol* **45**, 433–445 (2011).

25. Amann, B. *et al.* Spring temperature variability and eutrophication history inferred from sedimentary pigments in the varved sediments of lake żabińskie, north-eastern poland, AD 1907–2008. *Global and Planetary Change* **123**, 86–96 (2014).

26. Adl, S. M. *et al.* Revisions to the classification, nomenclature, and diversity of eukaryotes. *Journal of Eukaryotic Microbiology* **66**, 4–119 (2019).

27. Giguet-Covex, C. *et al.* Sedimentological and geochemical records of past trophic state and hypolimnetic anoxia in large, hard-water lake bourget, french alps. *J Paleolimnol* **43**, 171–190 (2010).

28. Alric, B. *et al.* Local forcings affect lake zooplankton vulnerability and response to climate warming. *Ecology* **94**, 2767–2780 (2013).

29. Belle, S., Verneaux, V., Mariet, A.-L. & Millet, L. Impact of eutrophication on the carbon stable-isotopic baseline of benthic invertebrates in two deep soft-water lakes. *Freshwater Biology* **62**, 1105–1115 (2017).

30. Sarazin, G., Michard, G., Gharib, I. A. & Bernat, M. Sedimentation rate and early diagenesis of particulate organic nitrogen and carbon in aydat lake (puy de dôme, france). *Chemical Geology* **98**, 307–316 (1992).

31. Lavrieux, M. *et al.* 6700 yr sedimentary record of climatic and anthropogenic signals in lake aydat (french massif central): *The Holocene* (2013) doi:10.1177/0959683613484616.

32. Nedjai, R., Nguyen-Trung, C. & Messaoud-Nacer, N. Multi-secular lead (pb) contamination on a regional scale: Comparative analysis of the grand-maclu and saint-point lakes in the jura area, france. *Journal of Advanced Chemical Engineering* **1**, 1–10 (2011).
