# Supplementary material for: Assessing the response of micro-eukaryotic diversity to the Great Acceleration using lake sedimentary DNA
Source: Nat Commun. 2020 Jul 31;11:3831. doi: 10.1038/s41467-020-17682-8 (PMC7395174; doi:10.1038/s41467-020-17682-8)
Supplement: Supplementary file 3 — Reporting Summary [file 41467_2020_17682_MOESM3_ESM.pdf]

nature research

Corresponding author(s): Domaizon Isabelle

Last updated by author(s): Jul 1, 2020

Reporting Summary

Nature Research wishes to improve the reproducibility of the work that we publish. This form provides structure for consistency and transparency in reporting. For further information on Nature Research policies, see our Editorial Policies and the Editorial Policy Checklist.

Statistics

For all statistical analyses, confirm that the following items are present in the figure legend, table legend, main text, or Methods section.

n/a	Confirmed	
☐	☒	The exact sample size (n) for each experimental group/condition, given as a discrete number and unit of measurement
☐	☒	A statement on whether measurements were taken from distinct samples or whether the same sample was measured repeatedly
☐	☒	The statistical test(s) used AND whether they are one- or two-sided<br>*Only common tests should be described solely by name; describe more complex techniques in the Methods section.*
☐	☒	A description of all covariates tested
☐	☒	A description of any assumptions or corrections, such as tests of normality and adjustment for multiple comparisons
☐	☒	A full description of the statistical parameters including central tendency (e.g. means) or other basic estimates (e.g. regression coefficient) AND variation (e.g. standard deviation) or associated estimates of uncertainty (e.g. confidence intervals)
☐	☒	For null hypothesis testing, the test statistic (e.g. F, t, r) with confidence intervals, effect sizes, degrees of freedom and P value noted<br>*Give P values as exact values whenever suitable.*
☒	☐	For Bayesian analysis, information on the choice of priors and Markov chain Monte Carlo settings
☒	☐	For hierarchical and complex designs, identification of the appropriate level for tests and full reporting of outcomes
☒	☐	Estimates of effect sizes (e.g. Cohen's d, Pearson's r), indicating how they were calculated

Our web collection on statistics for biologists contains articles on many of the points above.

Software and code

Policy information about availability of computer code

Data collection	No software was used for data collection
Data analysis	Bioinformatic analyses were performed using PANAM (v.0.94). Statistical analyses were performed with R v3.5.3 using package vegan (v.2.6-0) and DESeq2 (v.1.26.0). The R code to reproduce the analyses and results is available at https://doi.org/10.5281/zenodo.3662243

For manuscripts utilizing custom algorithms or software that are central to the research but not yet described in published literature, software must be made available to editors and reviewers. We strongly encourage code deposition in a community repository (e.g. GitHub). See the Nature Research guidelines for submitting code & software for further information.

Data

Policy information about availability of data

All manuscripts must include a data availability statement. This statement should provide the following information, where applicable:

- Accession codes, unique identifiers, or web links for publicly available datasets
- A list of figures that have associated raw data
- A description of any restrictions on data availability

All raw reads are available through ENA (https://www.ebi.ac.uk/ena) using the study accession number PRJEB35411. The taxonomic affiliation was performed using the Protist Ribosomal Reference database (PR2) available at https://pr2-database.org/. Any additional data needed to reproduce the results are available at https://doi.org/10.5281/zenodo.3662243.

Field-specific reporting

Please select the one below that is the best fit for your research. If you are not sure, read the appropriate sections before making your selection.

☐ Life sciences ☐ Behavioural & social sciences ☒ Ecological, evolutionary & environmental sciences

For a reference copy of the document with all sections, see nature.com/documents/nr-reporting-summary-flat.pdf

Ecological, evolutionary & environmental sciences study design

All studies must disclose on these points even when the disclosure is negative.

Study description	This paleolimnological study is based on the analysis of sedimentary DNA. The study reports the changes in micro-eukaryotic communities between the recent period (~2000) and the 19th century. Data are presented for 48 lakes. DNA metabarcoding (18S) was used to reconstruct micro-eukaryotic communities. Changes in community composition were investigated with two covariates: time (discrete variable 'top' vs. 'bottom') and elevetion (continuous variable). There is no experimental manipulation. Statistical analyses have been set up in such a way that they take account of the dependency within repeated measures (top and bottom for each lake).
Research sample	The 48 study sites were chosen because they cover different lake typologies and a wide altitude gradient, from lowland to high altitude lakes (see Supplementary Table 1).
Sampling strategy	To the authors' knowledge, the sample size is the largest to date for this type of research in molecular paleolimnology. The sample size is adequate to perform all the statistical analysis presented in this study with sufficient statistical power.
Data collection	Sediment coring was performed by LM, DR and DG in the deepest part of the lake basins using a UWITEC gravity corer.<br>The sub-sampling of sediment cores to isolate two sediment strata (corresonding to 2 key periods : 19th century and modern times) was performed by LM, ID, DE, DR.<br>Pigments analysis were performed by DE.<br>DNA extraction from sediment and DNA library preparation were performed by ID with the support of molecular biology labs organised for the analysis of sedimentary DNA (Thonon INRAE CARRTEL).<br>High throughput sequencing (MiSeq Technology) was performed by a national platform specialised on metagenomics (GeT-PlaGe Genotoul - Toulouse - FR).<br>Bioinformatics treatment was performed by DD and FK.<br>Data analysis was a collective work, led by FK and ID.
Timing and spatial scale	Field work was performed from 2010 to 2016 during spring and summer. Each lake was sampled once (sediment coring). The two sediment layers were sampled at the "top" and the "bottom" of each sediment record to document the two targeted periods, i.e. the modern times and the 19th century. The lakes sampled are located in France, within a 500km radius (see Supplementary Table 1 for geographical coordinates).
Data exclusions	From the 53 lakes initially sampled (Supplementary Table 1), 5 did not meet the requirements (quantity and quality of DNA extracted from sediments) for the reconstruction of micro-eukaryotic diversity and were excluded from the analyses.
Reproducibility	High throughput sequencing (HTS) was replicated 2 times for each sample. Technical HTS replicates exhibited a high degree of similarity (Supplementary Fig. 9) and were merged during the data processing.
Randomization	Not relevant: in this study there is no allocation of samples/individuals into groups.
Blinding	Not relevant: in this study there is no allocation of samples/individuals into groups that could be obfuscated.

Did the study involve field work? ☒ Yes ☐ No

Field work, collection and transport

Field conditions	The field work campaigns were carried out in good conditions to perform the samplings of sediment cores, i.e. favorable weather conditions (no rain and no wind).
Location	Geographical coordinates and altitude are provided for each lake in Supplementary Table 1.
Access & import/export	All the field work campaigns were carried out in compliance with local, national and international laws.
Disturbance	No particular disturbance was caused by this project.

Reporting for specific materials, systems and methods

We require information from authors about some types of materials, experimental systems and methods used in many studies. Here, indicate whether each material, system or method listed is relevant to your study. If you are not sure if a list item applies to your research, read the appropriate section before selecting a response.

Materials & experimental systems

n/a	Involved in the study
☒	☐ Antibodies
☒	☐ Eukaryotic cell lines
☒	☐ Palaeontology and archaeology
☒	☐ Animals and other organisms
☒	☐ Human research participants
☒	☐ Clinical data
☒	☐ Dual use research of concern

Methods

n/a	Involved in the study
☒	☐ ChIP-seq
☒	☐ Flow cytometry
☒	☐ MRI-based neuroimaging
