# Supplementary material for: Assessing the response of micro-eukaryotic diversity to the Great Acceleration using lake sedimentary DNA
Source: Nat Commun. 2020 Jul 31;11:3831. doi: 10.1038/s41467-020-17682-8 (PMC7395174; doi:10.1038/s41467-020-17682-8)
Supplement: Supplementary file 4 — Description of Additional Supplementary Files [file 41467_2020_17682_MOESM4_ESM.pdf]

Description of Additional Supplementary Files

File Name: Supplementary Data 1
Description: Complete list of OTUs used in the analyses with their representative DNA sequence, their taxonomic classification and their trophic classification
